# Buccal microRNA dysregulation in lung field carcinogenesis: Gender-specific implications

**DOI:** 10.3892/ijo.2014.2495

**Published:** 2014-06-11

**Authors:** RAMESH K. WALI, THOMAS A. HENSING, DANIEL W. RAY, MART DELA CRUZ, ASHISH K. TIWARI, ANDREW RADOSEVICH, LISA JEPEAL, HIRAN C. FERNANDO, VIRGINIA R. LITLE, MARJORY CHARLOT, NAVNEET MOMI, VADIM BACKMAN, HEMANT K. ROY

**Affiliations:** 1 Department of Medicine, Boston University Medical Center, Boston, MA 02118, USA; 2 Department of Surgery, Boston University Medical Center, Boston, MA 02118, USA; 3 Department of Surgery, NorthShore University Health Systems, Evanston, IL 60201, USA; 4 Department of Medicine, NorthShore University Health Systems, Evanston, IL 60201, USA; 5 Department of Medicine, Michigan State University, East Lansing, MI 48824, USA; 6 Department of Biomedical Engineering, Northwestern University, Evanston, IL 60201, USA

**Keywords:** human lung cancer, microRNA, buccal mucosa, biomarker, gender

## Abstract

MicroRNAs (miRNAs) have been shown to be reliable early biomarkers in a variety of cancers including that of lung. We ascertained whether the biomarker potential of miRNAs could be validated in microscopically normal and easily accessible buccal epithelial brushings from cigarette smokers as a consequence of lung cancer linked ‘field carcinogenesis’. We found that compared to neoplasia-free subjects, a panel of 68 miRNAs were upregulated and 3 downregulated in the normal appearing buccal mucosal cells collected from patients harboring lung cancer (n=76). The performance characteristics of selected miRNAs (with ≥1-fold change) were excellent with an average under the receiver operator characteristic curve (AUROC) of >0.80. Several miRNAs also displayed gender specificity between the groups. These results provide the first proof-of-concept scenario in which minimally intrusive cheek brushings could provide an initial screening tool in a large at-risk population.

## Introduction

Lung cancer ranks as the leading cause of cancer deaths in the United States with an estimated 224,210 new cases and 159,260 deaths projected in 2014 (1). The 5-year survival for lung cancer remains dismal at ~16% (2). The poor prognosis can be partly attributed to more than half of patients presenting with advanced stage at the time of diagnosis. This underscores the need for screening of the asymptomatic at-risk population. This may be feasible because ~85–90% of lung cancers occur in a readily identifiable population e.g., smokers, etc. (3). However, previous large scale randomized trials utilizing chest X-ray or sputum cytology have not yielded satisfactory results in lung cancer screening (4). Recently the National Lung Screening Trial (NLST) garnered considerable excitement with the demonstration that low dose computed tomography (LDCT) lowered lung cancer mortality by ~20% (95% Cl, 6.8–26.7; p=0.004) (5,6). This was the impetus for number of groups including the US Preventive Services Task force (USPSTF) to recommend screening high risk populations (defined as age range of 55–74 years, smoking ≥30-pack-years, and if quit, must be for >15 years) (7). Unfortunately, the specificity for lung cancer was only 73.4% and this coupled with the low prevalence of malignancy (1.1%) lead to dismal positive predictive value with >95% of positives being false positives (4). Thus, to mitigate the harm from these false positives, it is critical to enrich the proportion from those harboring lung cancer to identify a group that would be more appropriate for screening. Therefore, to minimize unnecessary radiation exposure, expense and false positives from LDCT, it is essential to pre-identify patients for any benefit via relatively simple and minimally intrusive pre-screen examinations.

There has been some interest in exploring buccal (cheek) mucosa as an extended field carcinogenesis site for lung cancer (8). Indeed, it is increasingly clear that buccal mucosa is in the ‘field of injury’ of tobacco smoke (9) and considered to be a ‘molecular mirror’ of lung carcinogenesis (10). Interestingly, malignancy related changes have been reported earlier in the epithelial cells collected from normal buccal mucosa from lung and breast cancer patients (11). To date, a myriad of genes have been reported to be dysregulated in the microscopically normal buccal mucosa of patients with lung cancer (12,13). Thus exploitation of buccal mucosa for lung cancer screening and chemoprevention may be a promising undertaking. However, finding a reliable biomarker in the buccal mucosa that can effectively gauge lung cancer associated field carcinogenesis has been challenging.

MicroRNAs have received increasing interest as biomarkers in a variety of cancers. These short, non-coding RNA molecules are typically 17–22 nucleotides in length and impact gene expression through decreasing mRNA stability and/or blocking translation (14). Thus, they can act as powerful effectors in both early and late lung carcinogenesis. Studies have shown that not only can miRNAs help in sub-classifying different stages of non-small cell lung carcinomas (NSCLC) (15) but specific miRNA profiles could also predict disease recurrence (16) and prognosis (17). While miRNA modulations are well established in the pathogenesis of lung cancer (as recently reviewed by our group) (18), to date no report suggests their role in field carcinogenesis (19). Furthermore, while there is a plethora of data suggesting that lung cancer in men and women are biologically distinct, scarce data are available on gender-specific biomarkers (20). This leads us to hypothesize that buccal miRNA expression has the potential to be a dependable biomarker of lung cancer and may define gender-specific differences.

## Materials and methods

### Clinical sample collection

In this case-control study, controls were current or former smokers who were recruited from either a chronic obstructive pulmonary disease (COPD) clinics or other non-pulmonary clinic without a history of lung cancer. Cases were smokers (current/past) with pathologically diagnosed lung cancer prior to chemo-radiation therapy. All studies were performed under the Institutional Review Board supervision.

### Sample preparation

Mucosal brushings from a visually normal buccal cheek surface were obtained with a cytology brush (CytoSoft #Cyb-1; Medical Packaging Corp., Camarillo, CA, USA). The cells were collected, applied to a glass slide and fixed with 70% ethanol followed by air drying and storage at −80°C. Total cellular RNA was isolated using Ribopure RNA kit (Ambion) following the manufacturer’s instructions.

### Buccal mucosa miRNA profiling

After establishing RNA purity (OD ratio of 260/280), the samples were subjected to reverse transcription using MegaPlex RT Primers and TaqMan miRNA reverse transcription kits (Applied Biosystems, Foster City, CA, USA) following the manufacturer’s instructions. The cDNA was diluted in Universal PCR master mix-II (Applied Biosystems) and then loaded on to TaqMan^®^ Low Density Array (TLDA) microfluidic MicroRNA 384-well cards (Applied Biosystems) for real-time PCR (ABI 7900 HT RT-PCR System). The relative concentration of miRNAs was calculated by comparative (RQ = 2^−ΔΔCt^) analysis and the fold change determined as log10 RQ. The data were analyzed using RQ Manager 12.1 (Applied Biosystems) and RealTime StatMiner software (Integromics, Philadelphia, PA, USA).

### Statistical analysis

Statistical significance for the individual miRNA expression was performed using appropriate statistical tools from Microsoft Excel 2010 and the area under the receiver operator curve (AUROC) was calculated using STATA 8 software. A two tailed Student’s t-test was performed with attention to the false discovery rate (FDR).

## Results

### Demographic characteristics

We recruited 76 subjects for this study (39 controls and 37 cases of NSCLC). The majority of the controls had chronic obstructive lung disease (COPD) so as to closely match demographics, especially intensity of smoking, albeit we realize that COPD is an independent risk factor for lung cancer and may obscure some of our effect. Majority of NSCLCs were adenocarcinomas and the population was >80% white (Table I). The cases and controls were reasonably matched with cancer patients being slightly older and with higher smoking intensity (pack years) than the controls (Table I).

### MicroRNA modulation in the buccal mucosa of lung cancer patients

**Figure 1:**
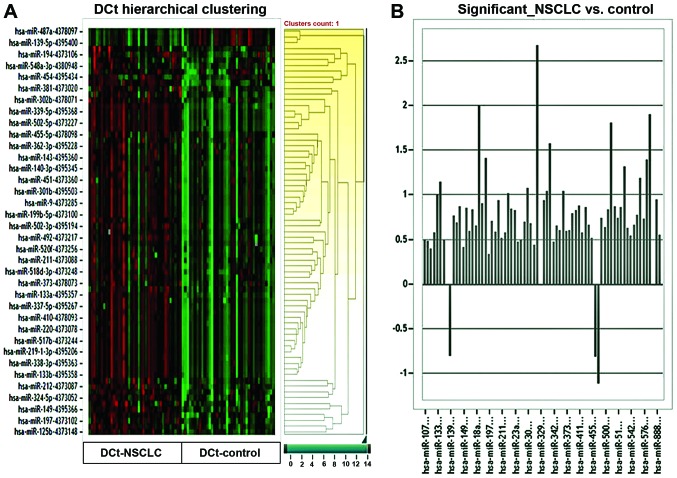
(A) Hierarchical clustering of normalized cycle threshold (DCt) values of differentially regulated miRNA in the buccal mucosa of NSCLC patients. (B) Histogram showing fold change of differentially expressed miRNA in the buccal mucosa collected from control and NSCLC patients. The change was calculated as log10 of relative quantitation.

To demonstrate characteristics of lung cancer field carcinogenesis in individuals at risk with concurrent lung cancer, we performed miRNA profiling of the buccal mucosa (extended field) collected from control and lung cancer patients. The heat map of normalized cycle threshold (DCt) values demonstrates significant differential upregulation of a panel of 68 miRNAs and downregulation of 3 miRNAs in the buccal mucosa collected from lung cancer cases compared to control (Fig. 1A). As shown in the histogram (Fig. 1B), the level of miRNA modulation (fold change) ranged from −1.27 to 2.85 as calculated from log10 RQ, where RQ is 2^−ΔΔCt^. We found twelve buccal miRNAs to be significantly altered between the groups (fold change of miR-23a = 1.25, miR-181c = 1.5, miR-192 = 1.72, miR-194 = 1.78, miR-208 = 1.2, miR-337-5p = 2.64, miR-338-3p = 1.32, miR-487a = −1.27, miR 502-5p = 2.85, miR-542-3p = 1.28, miR-628-5p = 1.82 and miR-672 = 1.86). The data represent a panel of miRNA that showed >1-fold difference (p≤0.05) between NSCLC and control subjects.

### Gender-specific differences in dysregulated buccal microRNAs

**Figure 2:**
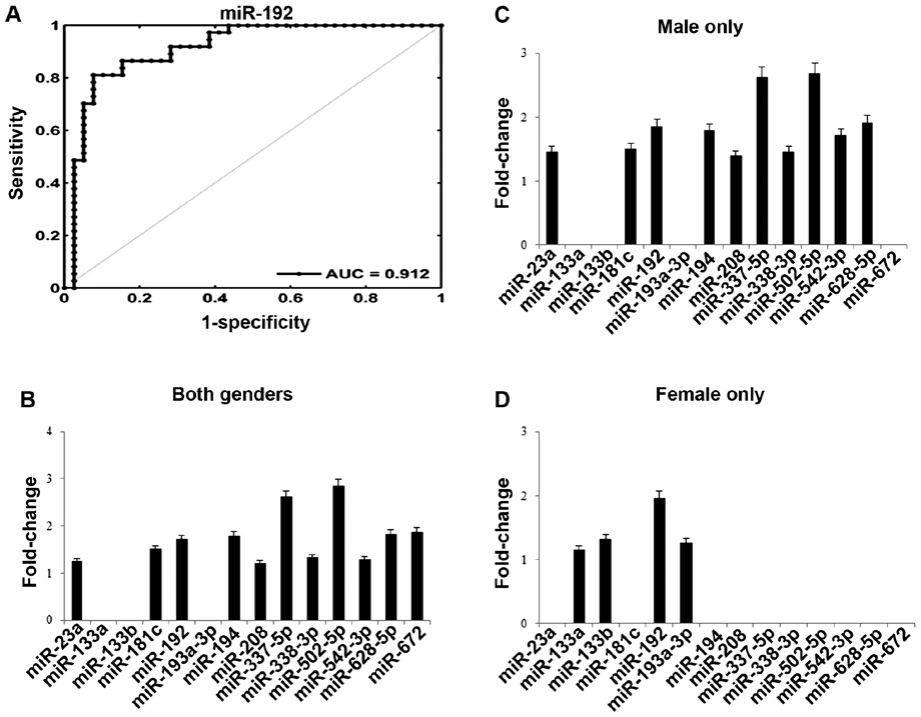
Lung cancer prediction and gender specificity. (A) AUROC curve for one representative microRNA (miR-192) showing discrimination between smokers without cancer (COPD) with those having lung cancer. (B–D) Selected miRNAs from the buccal mucosa that showed excellent power to discriminate between control and cancer patients (average AUROC values ≥0.8). The data depict miRNA that showed >1-fold difference between NSCLC and control subjects (p<0.05). In both genders, out of 68 miRNAs differentially expressed, only 11 were identified to have AUROC of ≥0.8 with excellent power to discriminate between control and lung cancer patients (B). In terms of gender specificity a set of 10 miRNAs for males (C) and 4 miRNAs for females (D) were found to be significant (p≤0.05).

Based on the above data, differential expression of a panel of buccal miRNA provides an important marker for field carcinogenesis with respect to concurrent lung neoplasia. To understand whether alterations in the identified miRNA markers in the buccal mucosa could also be utilized to predict future neoplasia, we performed receiver operator characteristic curve analysis (AUROC) of the miRNA DCt values using STATA program (Fig. 2A, representative AUROC; miR-192). A set of 11 miRNAs could discriminate between smokers without cancer (COPD) and with those having lung cancer (both genders) with excellent predictive ability (AUROC >0.8; Fig. 2B). Furthermore, to understand any gender-specific predictability, we evaluated responses of these differential miRNA expressions to gender. Ten miRNAs in male NSCLC patients (Fig. 2C) and 4 miRNAs in female NSCLC patients (Fig. 2D) were differentially expressed compared to gender-specific controls. Out of all these miRNA panels, miRNA-192 was the only miRNA to be significantly altered in both males and females. Performance characteristics discriminating control from cases on the basis of gender is presented in Table II. Number of miRNAs demonstrated higher sensitivity (70–90%) between the gender groups when compared at a fixed specificity of 80% (Table II). All these gender-specific miRNAs had an excellent predictive ability to discriminate between control and cancer (AUROC >0.8).

## Discussion

We demonstrate, for the first time, that miRNA expression is differentially expressed in the normal buccal epithelium of patients harboring lung cancer and thus has potential to serve as a surrogate biomarker of an extended field of lung carcinogenesis. The preliminary estimate of performance characteristics appear promising with a panel of 11 miRNAs having robust ability to discriminate between cases and controls (AUROC >0.8). However, it appears critical to segregate these biomarkers in terms of gender as the majority appears to be gender-specific.

The field of injury concept is well recognized in lung cancer given that the entire aero-digestive tract is subjected to the deleterious effects of inhaled cigarette smoke (21). Indeed, this has clinically been used to evaluate higher risk of developing second primaries in lung cancer patients (screening for head and neck and esophageal cancer) (22–24). Despite being histologically normal, bronchial mucosa from lung cancer patients have been reported to exhibit diffuse p53 mutations (25). Similarly, deregulation of PI3K pathway has also been reported in the bronchial airway epithelium of smokers as an early event in lung carcinogenesis (26). In a number of gene expression trials, microscopically normal bronchial epithelium from patients harboring lung cancer has been shown to possess altered transcriptome (27). Importantly, while some of these changes are reversible others are permanent, consonant with the long-term risk of lung cancer even after smoking cessation (28). From a diagnostic point of view, the study of Spira and colleagues was pivotal for having identified 80 gene biomarkers from the microscopically normal right mainstem bronchus that could discriminate between smokers with and without lung cancer with 80% sensitivity and 84% specificity (29). Others have noted that a key 14 gene (anti-oxidant, DNA repair and transcription) signature in the normal airway epithelium had comparable biomarker performance characteristics (30).

As discussed earlier, the oropharyngeal epithelium is susceptible to field of injury as a result of tobacco smoke. Several studies have demonstrated that buccal mucosal gene expression mirrors bronchial dysregulations (10) and therefore, may provide an easily accessible resource to evaluate field of injury. Lung and breast cancers have been reported to induce few selected malignancy associated nuclear features that may offer tools for cancer screening (11). Similarly, microsatellite analysis has revealed differences in the loss of heterozygosity (LOH) in exfoliated oral mucosal cells between smoking and non-smoking cancer patients (31). However, due to the presence of active salivary RNAses in the oral cavity, RNAs collected from the buccal mucosa are prone to a higher rate of degradation during collection thus causing a vexing problem for gene expression analyses (12). In fact, for these reasons nasal mucosa has been shown to perform better than buccal mucosa (32). Therefore, for buccal biomarkers to be more robust than the labile mRNA, recently, it has become apparent that small non-coding miRNAs are more robust and resistant to degradation (33). Numerous groups, including our own, have shown that miRNA dysregulation can be marker of field carcinogenesis in a variety of organs (colon, ovary, etc.) (34,35). The role of miRNAs in lung cancer is well established. For instance, a number of studies have previously reported dysregulation of miRNA in lung cancer (19) and their involvement in the prognosis and responsiveness to therapy (36). Furthermore, assessment of miRNAs in the blood have been reported to provide useful tools in lung cancer screening and diagnosis but it is still unclear if they can discriminate stage I/II NSCLC from more advanced cancer (37).

To our knowledge, this is the first report demonstrating alterations in the expression of miRNA in the buccal mucosa collected from lung cancer patients. Importantly, some of the miRNAs identified in the buccal mucosa did appear to partially correlate with factors in the bronchial epithelium (38). However, in the buccal mucosa we observed a greater proportion of miRNAs to be upregulated than previously reported in miRNA profiles of serum (37) or bronchial tissues from lung cancer patients (39). There could be several reasons for these discrepancies including the variability and lack of reproducibility which is characteristic of the literature. The miRNA expression pattern may not remain the same during bronchial squamous carcinogenesis but evolve with time and with cancer staging (40). Furthermore, meta-analysis data comparing miRNA expression profiles in lung cancer tissues with those in normal tissues has revealed a number of inconsistencies from different studies (41). Several variations in miRNA have also been reported between tissue-based and plasma-based profiles with later being more tumor specific (42). Thus, it is important to note that as a result of expanded field of injury buccal mucosa may respond with a distinctive miRNA expression profile.

From a clinical perspective, the diagnostic characteristics appear to be quite promising. While caution needs to be applied, given this is a preliminary report, it has the potential to provide a pre-screen to identify a higher-cancer prevalent groups that may further need to undergo more expensive LDCT. This could be analogous to the two step paradigms established in other organs such as Pap smear → colposcopy for cervical cancer or fecal occult blood test (FOBT) → colonoscopy for colorectal cancer. It is important to note that despite modest sensitivities with both Pap smear and FOBT, these tests have been demonstrated to have a significant impact on mortality from these cancers.

With regards to gender, it is increasingly realized that lung cancer in men and women are somewhat distinct. In general, it takes less cigarette smoking for women to develop lung cancer than men (43). Furthermore, while the incidence of lung cancer is comparable between men and women (new cases in 2014; 116,000 males and 108,210 females), the incidence is decreasing in men but increasing in women (1). Women also tend to have molecularly distinct lung cancers (higher prevalence of epidermal growth factor receptor mutations) (44) and have a better prognosis (45). This concept has not been used in screening strategies. However, for another common gender neutral malignancy (colorectal) both blood based markers (e.g., c-reactive protein) (46) and markers of field carcinogenesis (e.g., microvascular) (47), we have previously demonstrated that biophotonic markers of field carcinogenesis had a gender-specific predilection. With regards to miRNA, in this study, we were able to identify 4 buccal specific miRNAs in female NSCLC patients that were differentially regulated compared to respective controls. The etiology for the differential effect is unclear but probably reflects the interaction between the exogenous stimuli (cigarette smoke) and the genetic makeup (response to insult). It is interesting to note that only ~10% of smokers will eventually develop lung cancer. Sex steroids have been shown to be somewhat protective but unlikely to play a role in these studies since most women were post-menopausal. One intriguing but highly speculative possibility is alterations in oral microbiota which is different in patients with systemic cancer (i.e., pancreatic) (48). Others have shown profound gender related alterations in microbiome that correspond to systemic disease (diabetes) with concomitant increase in testosterone (49). It bears emphasis that these are highly speculative but regardless of the etiology, it does not impugn the potential clinical relevance of buccal miRNAs as a biomarker for lung cancer.

There are several limitations to this study that need to be acknowledged. The small dataset precludes meaningful subgroup analysis for histological subtype or stage. Our pilot studies failed to note any histological subgroup difference and this is consistent with our biophotonics approach suggesting that buccal approaches may not be affected by these factors (50). Secondly, the TLDA microarray platform chosen was extensive but certainly not exhaustive. In addition, more empirical data could be obtained with newer techniques such as RNA-seq (51). Thirdly, there could be potential confounding by other aero-digestive malignancies (e.g., head and neck cancer, and esophageal squamous carcinoma); however, the sheer volume of lung cancer cases may dwarf any other malignancy. Fourthly in any discovery biomarker study, the risk of over-fitting for diagnostic claims is significant and thus we are very circumspect until future studies with independent validation sets are completed.

In conclusion, our report shows for the first time that specific miRNAs from the buccal mucosa are altered in patients harboring lung cancer when compared with neoplasia-free smokers. Overall the number and magnitude of miRNAs altered was significant, emphasizing the potential clinical promise of this approach. Importantly, it is apparent that from a diagnostic perspective, generating separate signatures in males and females is mandatory for adequate discrimination. If confirmed in a larger dataset, this may herald the use of buccal miRNA biomarkers as a modality for risk stratification for determining which patients should undergo low dose CT.

## Figures and Tables

**Table I:** Patient characteristics.

Demographic information	Controls) (n=39	Cases (n=37)	p-value
Age (mean ± SD)	57±18	70±10	0.001
Race (% white)	84.6	83.8	-
Gender (% male)	66	52	-
Smoking (pack-years)	22±18	31±22	0.04

Total number of subjects from whom buccal brushings were collected was 76. The average age of patients in this study with cancer was statistically higher than without cancer.

**Table II:** Performance characteristics (% sensitivity) of buccal miRNAs for discriminating between control (no neoplasia) and cases (those harboring lung cancer): gender effect.

MiRNA	Both genders	Males only	Females only
MiR-23a	72	72	-
MiR-133a	-	-	85
MiR-133b	-	-	76
MiR-181c	74	88	-
MiR-192	87	82	88
MiR-193a-3p	-	-	74
miR-194	72	72	-
miR-208	66	74	-
miR-337-5p	78	74	-
miR-338-3p	75	75	-
miR-502-5p	98	90	-
miR-542-3p	70	74	-
miR-628-5p	66	78	-
miR-672	97	-	-

The sensitivity was calculated at 80% specificity for all the groups. All the miRNAs listed had an excellent predictive value (AUROC >0.8). There were 26 males and 13 females in the control group while the cases had 19 males and 18 females. The performance characteristics were compared between lung cancer and control subjects from ‘both genders’, ‘males only’ and ‘females only’ groups respectively. The miRNA identified had a fold increase of ≥1 between the cancer and non-cancer groups. The blank areas denote miRNA with poor performance characteristics (<60%).
